# Eye-Tracking Biomarkers and Autism Diagnosis in Primary Care

**DOI:** 10.1001/jamanetworkopen.2024.11190

**Published:** 2024-05-14

**Authors:** Brandon Keehn, Patrick Monahan, Brett Enneking, Tybytha Ryan, Nancy Swigonski, Rebecca McNally Keehn

**Affiliations:** 1 Department of Speech, Language, and Hearing Sciences, Purdue University, West Lafayette, Indiana; 2 Department of Psychological Sciences, Purdue University, West Lafayette, Indiana; 3 Department of Biostatistics and Health Data Science, Indiana University School of Medicine, Indianapolis; 4 Department of Pediatrics, Indiana University School of Medicine, Indianapolis

## Abstract

**Importance:**

Finding effective and scalable solutions to address diagnostic delays and disparities in autism is a public health imperative. Approaches that integrate eye-tracking biomarkers into tiered community-based models of autism evaluation hold promise for addressing this problem.

**Objective:**

To determine whether a battery of eye-tracking biomarkers can reliably differentiate young children with and without autism in a community-referred sample collected during clinical evaluation in the primary care setting and to evaluate whether combining eye-tracking biomarkers with primary care practitioner (PCP) diagnosis and diagnostic certainty is associated with diagnostic outcome.

**Design, Setting, and Participants:**

Early Autism Evaluation (EAE) Hub system PCPs referred a consecutive sample of children to this prospective diagnostic study for blinded eye-tracking index test and follow-up expert evaluation from June 7, 2019, to September 23, 2022. Participants included 146 children (aged 14-48 months) consecutively referred by 7 EAE Hubs. Of 154 children enrolled, 146 provided usable data for at least 1 eye-tracking measure.

**Main Outcomes and Measures:**

The primary outcomes were sensitivity and specificity of a composite eye-tracking (ie, index) test, which was a consolidated measure based on significant eye-tracking indices, compared with reference standard expert clinical autism diagnosis. Secondary outcome measures were sensitivity and specificity of an integrated approach using an index test and PCP diagnosis and certainty.

**Results:**

Among 146 children (mean [SD] age, 2.6 [0.6] years; 104 [71%] male; 21 [14%] Hispanic or Latine and 96 [66%] non-Latine White; 102 [70%] with a reference standard autism diagnosis), 113 (77%) had concordant autism outcomes between the index (composite biomarker) and reference outcomes, with 77.5% sensitivity (95% CI, 68.4%-84.5%) and 77.3% specificity (95% CI, 63.0%-87.2%). When index diagnosis was based on the combination of a composite biomarker, PCP diagnosis, and diagnostic certainty, outcomes were concordant with reference standard for 114 of 127 cases (90%) with a sensitivity of 90.7% (95% CI, 83.3%-95.0%) and a specificity of 86.7% (95% CI, 70.3%-94.7%).

**Conclusions and Relevance:**

In this prospective diagnostic study, a composite eye-tracking biomarker was associated with a best-estimate clinical diagnosis of autism, and an integrated diagnostic model including PCP diagnosis and diagnostic certainty demonstrated improved sensitivity and specificity. These findings suggest that equipping PCPs with a multimethod diagnostic approach has the potential to substantially improve access to timely, accurate diagnosis in local communities.

## Key Points

**Question:**

Can a battery of eye-tracking measures accurately identify young children with autism, and does integrating biomarkers with primary care practitioner (PCP) diagnosis provide a method for improving diagnostic accuracy?

**Findings:**

In this diagnostic study of 146 children aged 14 to 48 months, 6 eye-tracking indices were associated with reference standard autism outcome. A composite eye-tracking biomarker had 78% sensitivity and 77% specificity, and when integrated with PCP diagnosis and diagnostic certainty, had 91% sensitivity and 87% specificity.

**Meaning:**

These findings suggest that equipping PCPs with a validated, multimethod approach to autism evaluation has the potential to substantially improve access to timely, accurate diagnosis.

## Teaser

This diagnostic study evaluates whether a battery of eye-tracking biomarkers collected during clinical evaluation in the primary care setting can reliably differentiate community-referred young children with and without autism spectrum disorder.

## Introduction

In 2020, approximately 1 in 36 US children 8 years of age were diagnosed with autism.^1^ Although reliable identification can be made in the second year of life,^2^ the median age of autism diagnosis is after 4 years,^1^ with greater disparities for children from racial and ethnic minority backgrounds^3,4,5^ and underserved regions.^6^ Wait times for diagnostic evaluations often exceed a year,^7,8,9,10^ in part because the number of young children requiring an autism evaluation far exceeds available specialists.^11,12^ These delays impede engagement in early intensive evidence-based interventions, which support development at the time of optimal neuroplasticity and reduce long-term care costs.^13,14,15^

In light of the challenges in access to early autism diagnostic services, the development and evaluation of new models of community-based care delivery has been identified as a priority.^16^ For example, recent research has focused on training primary care practitioners (PCPs) to diagnose autism.^17,18,19,20,21,22^ However, given the complex, heterogeneous nature of the autism phenotype, multimethod community-based evaluation approaches that integrate both clinical and biobehavioral tools have the potential to improve the accuracy and timeliness of early diagnosis^16^ and reduce the lifetime costs associated with autism.^23^

To date, eye tracking is the most noninvasive, low-cost, and feasible approach to identifying early autism diagnostic biomarkers^24,25^ (ie, characteristics that provide discrete and objective indication of diagnosis^26^). Reliable differences in social vs nonsocial attention are present in autism,^27^ and pioneering work by Pierce and colleagues^28,29,30^ using the GeoPref test has shown nonsocial preference in a subset of young children diagnosed with autism. Jones and colleagues^31,32^ have also recently shown that reduced social engagement is a reliable diagnostic biomarker in autism. Additionally, prospective, longitudinal studies of infants at elevated likelihood for autism have also yielded potential biomarkers. These include less efficient nonsocial attentional disengagement,^33,34,35^ amplitude and latency differences of the pupillary light reflex (PLR),^36,37^ larger tonic baseline^38,39^ or resting^40^ pupil diameter, and differences in basic oculomotor measures, such as fixation duration^41^ and saccade amplitude.^42^ Although these are almost exclusively laboratory-based studies with effect sizes that suggest limited diagnostic utility,^43^ these results indicate that multiple eye-tracking indices may be combined to increase discriminative power^44^ and have promise for identifying autism in toddlers and young children.

The present study evaluated whether a battery of eye-tracking biomarkers collected during clinical evaluation in the primary care setting can reliably differentiate community-referred young children with and without autism. Further, we sought to determine whether combining multiple eye-tracking assays of autism likelihood together with PCP diagnosis and diagnostic certainty confers advantage for predicting diagnostic outcome in the community primary care setting.

## Methods

This diagnostic study was part of a larger project evaluating the diagnostic accuracy across the Early Autism Evaluation (EAE) Hub system, a statewide network of community PCPs trained to provide streamlined diagnostic assessment of children aged 14 to 48 months referred for autism evaluation.^45^ Primary care practitioners in the EAE Hubs follow a standard evaluation protocol and then issue a best-estimate autism diagnosis and report with clinical recommendations (additional details are provided in the eMethods in Supplement 1). This study was approved by the Indiana University School of Medicine Institutional Review Board, and written informed consent was obtained from legal guardians of all participants. The study followed the Standards for Reporting Diagnostic Accuracy (STARD) reporting guideline. 

### Participants

**Figure 1.**
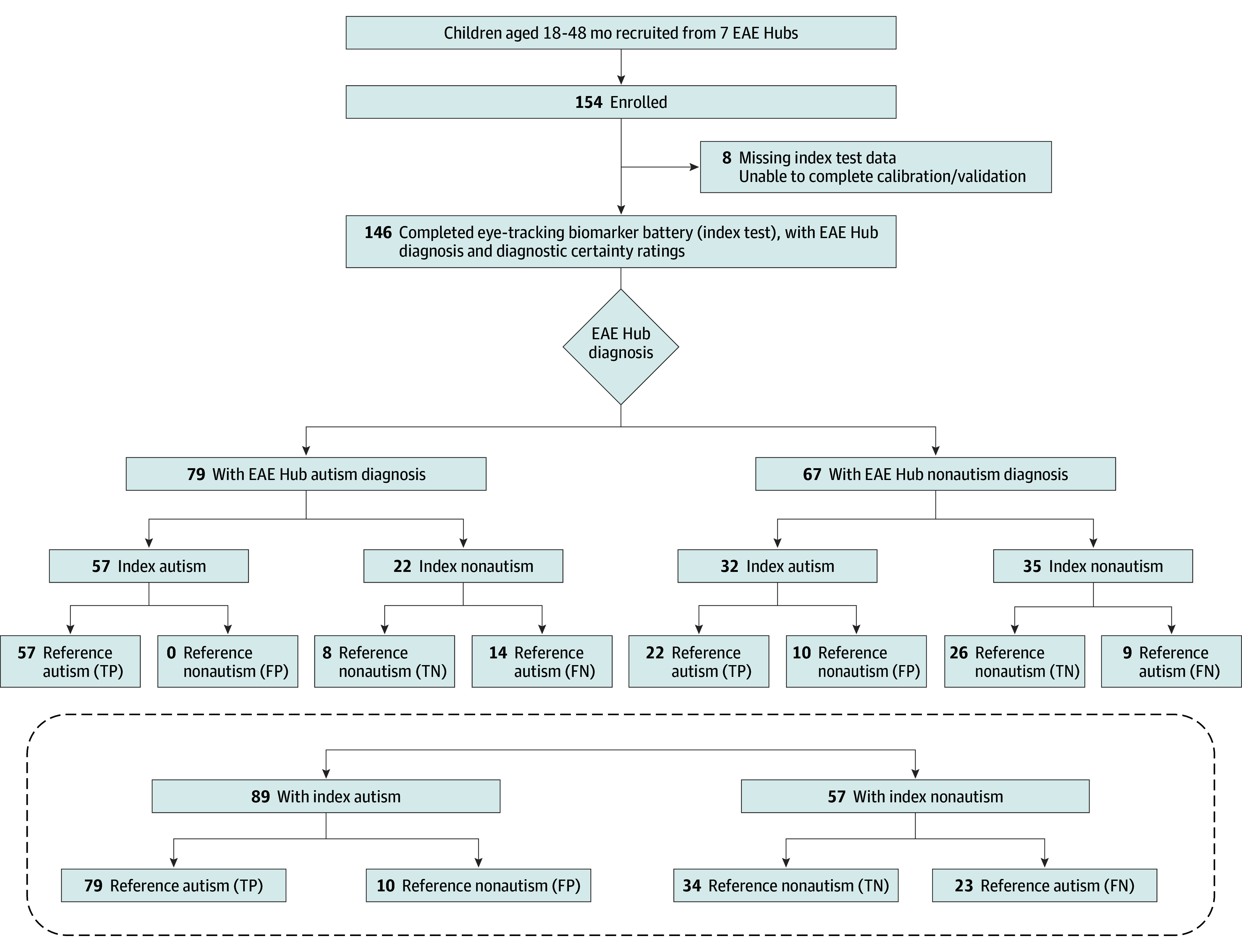
Participant Enrollment and Outcomes Flowchart After the Early Autism Evaluation (EAE) Hub visit and diagnosis, enrolled children completed a second visit to confirm reference standard diagnosis (autism or nonautism), which was made by a licensed clinical psychologist based on standardized assessment, as well as index diagnosis (autism or nonautism) based on a composite eye-tracking biomarker. For each participant, EAE Hub diagnosis (which could differ from the reference standard diagnosis) is included as a separate outcome measure. The lower portion of flowchart illustrates index and reference standard outcome irrespective of EAE Hub diagnosis. FN indicates false negative; FP, false positive; TN, true negative; and TP, true positive.

A total of 154 children evaluated in the EAE Hub system participated (Figure 1), with 146 (95%; reference standard autism group, 102; nonautism group, 44) providing usable data for at least 1 eye-tracking measure (eResults in Supplement 1 for details on missing and usable data). To be included, children were aged 14 to 48 months at time of EAE Hub evaluation and had an English-speaking primary caregiver or guardian.

### Study Design and Procedure

Seven EAE Hubs set within primary care practices referred children evaluated for autism to the study between June 7, 2019, and September 23, 2022. Each site referred a prospective, consecutive sample of children who received an EAE Hub evaluation. This recruitment procedure allowed the study team to maintain diagnostic blindness and assess children without referral bias. Once enrolled, an electronic survey was completed by the child’s caregiver (ie, child’s race and ethnicity and caregiver or family income and educational level) and a separate member of the study team collected autism evaluation data from the EAE Hub PCP (ie, autism or nonautism diagnosis and diagnostic certainty). Next, the study team traveled to the EAE Hub to conduct a follow-up criterion standard autism diagnostic assessment (reference standard diagnosis) and eye-tracking biomarker battery (index test) within 16 weeks of EAE Hub evaluation (eMethods in Supplement 1 provides more details).

### Reference Standard Diagnosis

Reference standard diagnosis (reference autism or nonautism) was made by a licensed clinical psychologist (B.E., T.R., and R.M.K.) with expertise in autism diagnosis. The diagnosis was based on a research evaluation including the Autism Diagnostic Observation Schedule, Second Edition (ADOS-2),^46^ Vineland Adaptive Behaviors Scale, Third Edition (VABS-3),^47^ Mullen Scales of Early Learning (MSEL),^48^ and a caregiver interview to assess for *Diagnostic and Statistical Manual of Mental Disorders*^49^ autism criteria (the eMethods in Supplement 1 provides a description of measures).

### EAE Hub Diagnosis and Diagnostic Certainty

The EAE Hub PCPs submitted categorical diagnosis (autism or nonautism) and diagnostic certainty (rated on a 5-point Likert scale as completely certain, highly certain, somewhat certain, slightly certain, or not at all certain). Levels of EAE Hub PCP certainty were dichotomized into certain (completely or highly certain) vs uncertain (somewhat, slightly, or not at all certain).

### Eye-Tracking

Participants first completed a 5-point calibration and validation procedure while viewing an animated cartoon with sounds. Prior to the start of each block, drift check and correction were completed. The eye-tracking biomarker battery included 5 paradigms: (1) an adapted GeoPref test modeled after that of Pierce et al^28,29,30^ to measure nonsocial preference (percentage of looking time to nonsocial video), (2) a gap-overlap paradigm to measure attentional disengagement (overlap-gap response time [RT] and no-shift percentage), (3) a PLR test similar to that of Nyström et al^36,37^ to measure PLR latency and amplitude, (4) a resting eye-tracking task to measure tonic pupil size and oculomotor metrics (fixation duration, saccade amplitude and duration), and (5) a passive visual exploration task to measure oculomotor metrics (eFigure and eMethods in Supplement 1 provide detailed descriptions). Eye-tracking quality control indices included accuracy, as measured by the displacement (in degrees visual angle) between the central fixation and location of gaze at each drift check, and precision, as measured by the root mean square of the Euclidean distances between samples during a fixation (for all participant fixations),^50^ with lower values indicative of better precision.

### Statistical Analyses

Data analyses were performed using JMP, version 13.0 (SAS Institute Inc). All analyses were tested at a 2-sided significance level of α = .05.

#### Biomarker Classification Accuracy

To examine whether eye-tracking biomarkers were associated with autism diagnostic outcome, a series of binary logistic regressions with eye-tracking indices as independent variables and reference standard autism diagnosis as the binary outcome were conducted. Analyses with unadjusted models were initially performed, and subsequent analyses with adjusted models were performed with age and sex included as covariates. Receiver operating characteristic curves and area under the curve (AUC) statistics were used to appraise discrimination of classification models. To assess the associations between individual eye-tracking metrics and clinical profiles, correlations were conducted between significant biomarkers and standardized clinical measures.

In addition, to combine significant individual biomarkers into a single metric, cutoffs for each measure were determined. Because of the substantial heterogeneity of the autism phenotype and overlapping symptoms with other neurodevelopmental disabilities,^26,51^ thresholds for biomarkers have been selected to increase specificity and decrease false-positive findings.^28^ Thus, for each biomarker, cut points were determined based on achieving 95% specificity, which yielded the best discrimination of reference standard diagnosis compared with alternative cut points that were explored. For each biomarker, sensitivity, specificity, positive predictive value (PPV), and negative predictive value (NPV) were calculated (eTable 6 in Supplement 1). Based on 95% specificity cutoff values, binary variables (0 indicates did not exceed threshold; 1, exceeded threshold) were created for each significant biomarker. Next, a dichotomous index test variable (biomarker-positive [index autism], biomarker-negative [index nonautism]) was created by combining the 6 individual biomarkers into a single composite metric. Specifically, a biomarker-positive score was given to any participant exceeding the 95% specificity cutoff for any of the 6 significant biomarkers. Missing data were not imputed; participants who did not provide usable data for a given task were assumed not to have exceeded the cutoff. Diagnostic groups did not differ in the number of missing individual biomarkers (eResults and eTable 1 in Supplement 1). Chance-corrected agreement (κ statistic) and accuracy indices of sensitivity, specificity, PPV, NPV, and their 95% CIs were reported for the composite biomarker (index test: biomarker-positive and biomarker-negative).

Pearson correlations, together with 95% CIs, were conducted to examine associations between each significant biomarker and standardized clinical measures (ADOS-2 Calibrated Severity Score, VABS-3 Adaptive Behavior Composite, and MSEL Early Learning Composite). For each significant biomarker, a familywise error rate of α = .05 was set, and a Bonferroni correction was used to conservatively account for inflated type I errors, with an α of 0.05 divided by 3 (ie, the number of standardized clinical measures examined), resulting in an adjusted threshold of .0167.

#### Biomarker Classification Accuracy With EAE Hub Diagnosis and Certainty

Next, to examine whether individual and composite eye-tracking biomarkers provided additional diagnostic utility beyond that provided by EAE Hub PCP diagnosis and diagnostic certainty, these variables as well as the interaction between diagnosis and certainty were included in logistic regression (with each biomarker in a separate model) to estimate reference standard autism diagnosis. The diagnosis × certainty interaction was included in the model, as previous research has shown that correct classification by PCPs is associated with diagnostic certainty.^45,52^

#### Classification and Regression Tree Analysis

To provide descriptive information complementary to logistic regression, a classification and regression tree (CART) analysis,^53^ based on recursive partitioning, was used to determine which combination of variables (EAE Hub PCP diagnosis, diagnostic certainty, composite biomarker, and biomarker frequency [sum of all individual biomarkers (0-6) that exceeded the 95% specificity threshold for each child]) were best predictive factors for reference standard autism diagnosis. This technique sequentially determines the variable that accounts for the greatest amount of variance in the outcome and partitions the sample at each node based on the most useful cutoff score. Furthermore, CART analysis offers the advantage of user-friendly interpretation by providing a graphical representation of the hierarchy of predictive factors, which may be critical as clinicians are likely to trust and use predictive rules that have face validity.^54^ Finally, because the goal of tiered diagnostic models, such as the EAE Hub system, is that PCPs can rule in or rule out autism in a subset of children and refer more complicated cases to a diagnostic specialist,^55^ terminal nodes for the CART analysis were identified as autism, nonautism, or refer for further evaluation.

## Results

### Biomarker Classification Accuracy

Among 146 children included in the analysis (mean [SD] age, 2.6 [0.6] years; 104 [71%] male and 42 [29%] female; 2 [0.1%] Asian, 14 [10%] Black, 21 [14%] Latine of any race, 96 [66%] non-Latine White, and 6 [4%] multiracial for those with data available; 102 [70%] with reference standard autism diagnosis) (Table 1), 6 eye-tracking measures were associated with an expert autism diagnostic outcome in both unadjusted and adjusted models, including nonsocial preference, no-shift percentage, PLR latency and amplitude, and resting and exploration fixation durations (Table 2 and eTable 2 in Supplement 1). Results from the adjusted models, which included age and sex, were reasonably consistent with unadjusted models for all 6 biomarkers.

**Table 1.** Participant Characteristics

Characteristic	Participant group		*P* value^a^
	Autism	Nonautism	
Total, No.	102	44	
Sex, No. (%)			
Male	78 (76)	26 (59)	.03
Female	24 (24)	18 (41)	
Age, mean (SD) [range], y	2.6 (0.6) [1.7-4.1]	2.4 (0.6) [1.7-4.0]	.12
MSEL ELC score, mean (SD) [range]	58 (10) [49-101]	77 (16) [49-114]	<.001
MSEL verbal DQ score, mean (SD) [range]	44 (20) [10-94]	76 (18) [28-109]	<.001
MSEL nonverbal DQ score, mean (SD) [range]	68 (16) [23-106]	85 (14) [44-124]	<.001
VABS-3 ABC score, mean (SD) [range]	65 (9) [30-88]	78 (11) [61-109]	<.001
ADOS-2 Comparison Score, mean (SD) [range]	9 (2) [3-10]	3 (2) [1-10]	<.001
Race and ethnicity, No. (%)			
Asian	2 (2)	0	.02
Black	11 (11)	3 (7)	
Latine, any race	20 (20)	1 (2)	
Non-Latine White	59 (58)	37 (84)	
Multiracial	5 (5)	1 (2)	
Unspecified or not reported	5 (5)	2 (5)	
Household income, No. (%)			
<$50 000	51 (50)	21 (48)	.40
$50 001-$99 999	25 (25)	16 (36)	
≥$100 000	16 (16)	5 (11)	
Unspecified or not reported	10 (10)	2 (5)	
Caregiver educational level, No. (%)			
High school diploma or less	33 (32)	17 (39)	.25
Some college or associate’s degree	34 (33)	19 (43)	
Bachelor’s degree	26 (25)	5 (11)	
Graduate degree	8 (8)	3 (7)	
Unspecified or not reported	1 (1)	0	
Other nonautism reference diagnoses, No. (%)			
GDD	84 (82)	17 (39)	<.001
LD	11 (11)	13 (30)	
Other^b^	1 (1)	3 (7)	
None	6 (6)	11 (25)	

Abbreviations: ABC, Adaptive Behavior Composite; ADOS-2, Autism Diagnostic Observation Schedule, Second Edition; DQ, Developmental Quotient; ELC, Early Learning Composite; GDD, global developmental delay; LD, language delay; MSEL, Mullen Scales of Early Learning; VABS-3, Vineland Adaptive Behaviors Scale, Third Edition.

^a^ Calculated using 2-sided *t* tests for continuous variables and χ^2^ tests for categorical variables.

^b^ Includes emotional behavioral concerns such as separation anxiety, sensory processing impairment, and parent-child relational problem.

**Table 2.** Biomarkers Significantly Associated With Reference Standard Autism Diagnosis

Predictive factors	Participant group, mean (SD) [range]		Unadjusted models				Adjusted models^a^		
	Autism	Nonautism	OR (95% CI)	Wald χ^2^ test	*P* value	AUC	OR (95% CI)	Wald χ^2^ test	*P* value
Nonsocial preference, %	46.20 (22.33) [3.35 to 86.80]	21.50 (12.48) [0.62 to 56.72]	1.07 (1.04 to 1.10)	25.8	<.001	0.82	1.07 (1.04 to 1.10)	23.7	<.001
No shift-gap effect, %	8.65 (13.42) [−25 to 50]	0.83 (10.45) [−16.67 to 40]	1.07 (1.01 to 1.12)	5.1	.02	0.72	1.07 (1.01 to 1.13)	4.9	.03
Resting fixation duration, ms	527.84 (135.71) [300 to 966]	460.93 (110.47) [239 to 686]	1.004 (1.001 to 1.008)	6.5	.01	0.63	1.01 (1.001 to 1.01)	6.9	.009
PLR latency, ms	268.51 (25.88) [207 to 338]	291.44 (23.62) [254 to 353]	0.96 (0.94 to 0.99)	7.1	.008	0.75	0.96 (0.93 to 0.99)	7.3	.007
PLR amplitude, %	32.69 (10.26) [10.17 to 57.60]	24.70 (8.23) [11.39 to 40.55]	1.10 (1.02 to 1.18)	5.9	.02	0.72	1.10 (1.01 to 1.19)	5.4	.02
Exploration fixation duration, ms	344.58 (63.10) [236 to 600]	320.80 (42.13) [232 to 402]	1.01 (1.00 to 1.02)	4.5	.03	0.61	1.01 (1.00 to 1.02)	6.6	.01

Abbreviations: AUC, area under the curve; OR, odds ratio; PLR, pupillary light reflex.

^a^ Adjusted for age and sex.

Correlational analyses between significant biomarkers and autism severity, developmental levels, and adaptive skills showed that nonsocial preference percentage was associated with decreased MSEL Early Learning Composite (*r* = −0.26 [95% CI, −0.44 to −0.06]) and VABS-3 Adaptive Behavior Composite (*r* = −0.31 [95% CI, −0.48 to −0.11]) scores for the reference standard autism but not nonautism groups (eTable 3 in Supplement 1). No other biomarkers were correlated with standardized measures in the autism or nonautism groups.

Individual biomarkers were not associated with each other, except for identical concepts examined in 2 tasks (ie, fixation duration) (eTable 5 in Supplement 1). Consistent with this, among children with a reference diagnosis of autism identified using the composite biomarker, most were identified with a single, unique biomarker (biomarker frequency of 1) (eTable 4 in Supplement 1). The mean (SD) number of drift checks over the course of the entire battery did not differ between autism (19 [10]) and nonautism (17 [8]) groups (*t*_144_ = −1.47 [*P* = .14]). Mean (SD) drift error (ie, accuracy) did not differ between autism (1.03 [0.38]) and nonautism (1.08 [0.26]) groups (*t*_141_ = 0.85 [*P* = .40]) across drift checks. Additionally, the mean (SD) variability of these offsets, as measured by the intraindividual SD of the offset measurements, did not differ between the autism (0.62 [0.99]) and the nonautism (0.60 [0.45]) groups (*t*_141_ = −0.15 [*P* = .88]). Mean (SD) precision values (root mean square of the sample-to-sample distances) did not differ between the autism (0.70 [0.35]) and the nonautism (0.69 [0.28]) groups (*t*_144_ = −0.13 [*P* = .90]).

### Biomarker Classification Accuracy With EAE Hub Diagnosis and Certainty

Predictive biomarkers were then tested in a model that included EAE Hub diagnosis and diagnostic certainty as well as their interaction associated with reference standard outcome. Whereas EAE Hub diagnosis was significant for most models, diagnostic certainty was not significant in any models; however, the EAE Hub diagnosis by certainty interaction was significant in all models, indicating that the main associations of EAE Hub diagnosis and certainty should be interpreted with caution, given that the ability of the EAE Hub diagnosis to estimate expert diagnosis depends on PCP diagnostic certainty.^45^ Importantly, all 6 biomarkers (including the composite biomarker) remained significant predictive factors of reference standard autism outcome after adjusting for these covariates (eTable 7 in Supplement 1).

### Agreement Between Index and Reference Diagnosis

For the composite biomarker, outcomes were concordant with the reference diagnosis for 113 cases (77%). Overall, 79 cases (54%) had true-positive findings and 34 (23%) had true-negative findings; 23 (16%) had false-negative findings and 10 (7%) had false-positive findings. Chance-corrected diagnostic agreement was moderate (κ = 0.51 [95% CI, 0.36-0.65]). Correct classification of autism (sensitivity) was 77.5% (95% CI, 68.4%-84.5%) and correct classification of nonautism (specificity) was 77.3% (95% CI, 63.0%-87.2%); PPV was 88.8% (95% CI, 80.5%-93.8%) and NPV was 59.6% (95% CI, 46.7%-71.4%).

### Classification and Regression Tree Analysis

**Figure 2.**
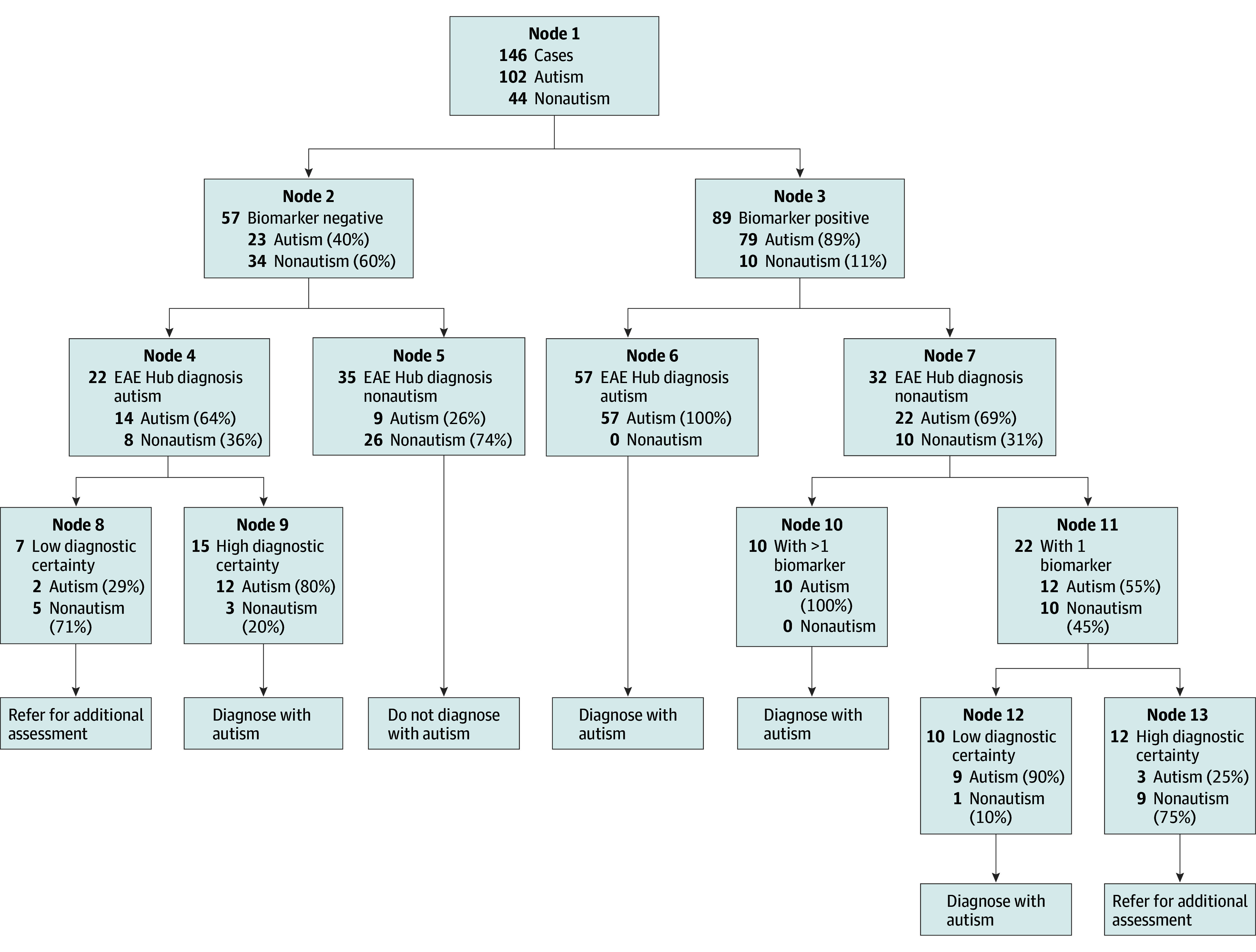
Decision Tree From Classification and Regression Tree Analysis At each split, the number of reference standard autism and nonautism cases and the percentage of the total cases within each split are presented. Final decisions (diagnose with autism, do not diagnose with autism, and refer) are displayed after each terminal node. EAE indicates Early Autism Evaluation.

Results of the CART analysis are shown in Figure 2. Tree growth was manually stopped after 5 splits, as additional splits did not improve the overall model fit (AUC, 0.92 [95% CI, 0.86-0.96]). The strongest baseline predictive factor of reference standard autism outcome was the composite biomarker, for which 79 of 89 (89%) with a biomarker-positive score had an autism outcome (node 3). Within the biomarker-positive group, 57 of 57 (100%) who were diagnosed with autism by the EAE Hub had a reference standard diagnosis of autism (node 6). Importantly, 22 of 32 cases (69%) with a nonautism EAE Hub diagnosis also had a reference standard diagnosis of autism (ie, false-negative findings; node 7). Within these cases, when more than 1 positive biomarker was present, 10 of 10 (100%) had a reference standard diagnosis of autism demonstrating that more than 1 positive biomarker identified autism cases when the EAE Hub PCP did not (node 10). Furthermore, when EAE Hub PCP certainty was low for nonautism cases, 9 of 10 cases (90%) in the biomarker-positive group also had a reference standard autism diagnosis (node 12). When the certainty of the EAE Hub nonautism diagnosis was high, 9 of 12 cases (75%) in the biomarker-positive group had a nonautism reference standard diagnosis (node 13).

Alternatively, when the composite biomarker was negative, 34 of 57 cases (60%) had a reference standard diagnosis of nonautism (node 2). Of those biomarker-negative cases, when autism was ruled out by the EAE Hub PCP, 26 of 35 (74%) did not have autism based on the reference standard (node 5). Of those biomarker-negative cases with an EAE Hub autism diagnosis, 14 of 22 (64%) had a reference standard diagnosis of autism (node 4). In those cases, if the EAE Hub autism diagnosis was highly certain, biomarker-negative cases were more likely to have reference standard diagnosis of autism (12 of 15 [80%]; node 9) compared with when certainty was low (2 of 7 [29%]; node 8).

Based on the CART analysis, diagnostic decisions of autism, nonautism, and referral for additional assessment were selected, resulting (when compared with reference standard diagnosis) in consistent outcomes for 114 of 127 cases (90%). Chance-corrected diagnostic agreement was substantial (κ = 0.73 [95% CI, 0.60-0.89]) with 90.7% sensitivity (95% CI, 83.3%-95.0%), 86.7% specificity (95% CI, 70.3%-94.7%), PPV of 95.7% (95% CI, 89.3%-98.3%), and a NPV of 74.3% (95% CI, 57.9%-85.8%).

A 5-fold cross-validation analysis confirmed good performance for the selected model. Specifically, the presented model was selected in 3 of 5 CART training runs, with 2 slightly different models selected in the remaining 2 CART training runs, indicating reasonably good model stability. Furthermore, the mean AUC for the 3 training runs that selected our model was 0.93 and their mean cross-validation AUC was 0.90, which was slightly higher than the other 2 models identified, indicating superior performance for the selected model, and its mean cross-validated AUC (0.90) was very high, demonstrating excellent out-of-sample performance.

## Discussion

The present diagnostic study acquired eye-tracking measures associated with autism likelihood in a diverse sample of young children evaluated for autism in the primary care setting. To our knowledge, this is the first study to collect eye-tracking biomarkers as part of community-based primary care autism diagnostic evaluation. Six eye-tracking indices differentiated children with autism from children with other developmental disabilities and provided unique information above and beyond PCP-based autism diagnosis and certainty. Our integrated CART diagnostic model demonstrated 90.7% sensitivity and 86.7% specificity.

### Eye-Tracking Diagnostic Biomarkers Classification of Autism in a High-Risk Community-Referred Sample

Given the phenotypic heterogeneity associated with autism, a single biomarker, especially one focused on reducing false-positive findings, is unlikely to capture all children on the autism spectrum. Therefore, the present study acquired multiple biomarkers with the goal of identifying unique cases among discrete markers. Our results are consistent with previous work indicating that nonsocial preference^28,29,30^ is a potent biomarker for identifying autism in young children. Additionally, findings of impairments in nonsocial attentional disengagement and shorter latency and increased amplitude of the PLR are consistent with previous research from other laboratory-based prospective longitudinal studies of infants at elevated likelihood for autism.^35,36,37^ Additionally, we found longer fixation durations for 2 distinct tasks in autism. This contrasts with previous findings of shorter fixation durations in infants later diagnosed with autism^41^; however, differences in child developmental levels and ages across these and other studies^35,40^ may explain differences across some biomarker metrics. Nevertheless, our biomarker composite (biomarker-positive) performed similarly to recent studies of eye-tracking–based measures used to identify autism in a specialty clinical setting^32^ and highlights the utility of these indices for identification of autism in a community-referred sample.

### Eye-Tracking Biomarkers and Additional Classification Accuracy Beyond PCP Diagnosis

While diagnostic biomarkers represent a novel tool to promote earlier and more accurate identification of autism, they are more likely to supplement clinical judgement rather than replace it.^32^ As such, if biomarkers do not contribute unique information in the diagnostic process, their utility is limited. The present study demonstrated that each individual biomarker, as well as the composite biomarker, contribute classification value beyond PCP diagnosis, certainty, and their interaction. In the context of autism diagnosis in the primary care setting, these biomarkers may give PCPs, who are not specialists in autism evaluation, further evidence necessary for accurate diagnostic decision making.

### Integrating Eye-Tracking Biomarkers Within Diagnostic Decision-Making

Approximately 30% to 40% of autism diagnoses made by clinicians, including PCPs in the present study, lack complete certainty.^56,57^ Results of the present study show that when incorporated into diagnostic decision-making, biomarkers may aid in identifying incorrect nonautism diagnoses (ie, misses), including those made with low certainty. For example, our CART model showed that 90% of children with an incorrect low certainty EAE Hub nonautism diagnosis were correctly identified based on having a positive composite biomarker (node 12 [Figure 2]). Thus, our results support an innovative method of autism diagnosis for young children in the primary care setting, which integrates clinical assessment by general pediatric PCPs with use of eye-tracking biomarkers to improve diagnostic accuracy.

### Limitations

This study has some limitations. Although confirmed using 5-fold cross-validation, the CART model was developed with the full dataset; future research with a larger sample is needed to assess the generalizability and performance of the model in other datasets. While we included a prospective, consecutively referred sample of children evaluated for autism, a class imbalance was present; such an imbalance has the potential to overinflate accuracy metrics,^58^ and thus our findings should be interpreted with caution. Although 95% of children provided at least 1 usable eye-tracking measure, the percentage of usable data across each task varied significantly (50%-94%) (eResults in Supplement 1). However, this is consistent with previous laboratory-acquired data for these paradigms,^30,59,60^ and no other group differences were present for quality control metrics.

## Conclusions

At the population health level, equipping PCPs with a validated, multimethod approach to early diagnosis has the potential to substantially improve access to timely, accurate diagnosis in local communities where the availability of neurodevelopmental experts is scarce. The findings of this diagnostic study suggest that multiple eye-tracking indices may be sensitive to autism, provide additional information beyond PCP diagnostic outcome and certainty, and, when integrated with these measures, may facilitate more accurate autism diagnosis.

## Notes

Supplement 1. **eMethods.** Description of Clinical Measures, Eye-Tracking Paradigms, and Eye-Tracking Data Processing **eResults.** Eye-Tracking Usable Data and Additional Regression Results **eFigure.** Significant Eye-Tracking Biomarkers **eTable 1.** Frequency and Percentage of Participants With Missing Biomarkers **eTable 2.** Biomarkers Not Significantly Associated With Reference Standard Autism Spectrum Diagnosis **eTable 3.** Correlation Between Significant Eye-Tracking Measures and Autism Spectrum Disorder Severity, Developmental Levels, and Adaptive Skills **eTable 4.** Number of Participants Within Each Group Who Have 0 to 5 Positive Biomarkers **eTable 5.** Correlation Matrix of Significant Biomarkers for All Participants **eTable 6.** Sensitivity, Specificity, Positive Predictive Value (PPV), and Negative Predictive Value (NPV) for All Significant Biomarkers **eTable 7.** Multivariable Logistic Regression Models for Predicting Reference Standard Autism Spectrum Disorder Diagnosis
eReferences

Supplement 2.
Data Sharing Statement
