# Supplementary material for: Eye-Tracking Biomarkers and Autism Diagnosis in Primary Care
Source: JAMA Netw Open. 2024 May 14;7(5):e2411190. doi: 10.1001/jamanetworkopen.2024.11190 (PMC11094561; doi:10.1001/jamanetworkopen.2024.11190)
Supplement: Supplement 1. — eMethods. Description of Clinical Measures, Eye-Tracking Paradigms, and Eye-Tracking Data Processing eResults. Eye-Tracking Usable Data and Additional Regression Results eFigure. Significant Eye-Tracking Biomarkers eTable 1. Frequency and Percentage of Participants With Missing Biomarkers eTable 2. Biomarkers Not Significantly Associated With Reference Standard Autism Spectrum Diagnosis eTable 3. Correlation Between Significant Eye-Tracking Measures and Autism Spectrum Disorder Severity, Developmental Levels, and Adaptive Skills eTable 4. Number of Participants Within Each Group Who Have 0 to 5 Positive Biomarkers eTable 5. Correlation Matrix of Significant Biomarkers for All Participants eTable 6. Sensitivity, Specificity, Positive Predictive Value (PPV), and Negative Predictive Value (NPV) for All Significant Biomarkers eTable 7. Multivariable Logistic Regression Models for Predicting Reference Standard Autism Spectrum Disorder Diagnosis eReferences [file jamanetwopen-e2411190-s001.pdf]

Supplementary Online Content

Keehn B, Monahan P, Enneking B, Ryan T, Swigonski N, McNally Keehn R. Eye-tracking biomarkers and autism diagnosis in primary care. *JAMA Netw Open*. 2024;7(5):e2411190. doi:10.1001/jamanetworkopen.2024.11190

eMethods. Description of Clinical Measures, Eye-Tracking Paradigms, and Eye-Tracking Data Processing

eResults. Eye-Tracking Usable Data and Additional Regression Results

eFigure. Significant Eye-Tracking Biomarkers

eTable 1. Frequency and Percentage of Participants With Missing Biomarkers

eTable 2. Biomarkers Not Significantly Associated With Reference Standard Autism Spectrum Diagnosis

eTable 3. Correlation Between Significant Eye-Tracking Measures and Autism Spectrum Disorder Severity, Developmental Levels, and Adaptive Skills

eTable 4. Number of Participants Within Each Group Who Have 0 to 5 Positive Biomarkers

eTable 5. Correlation Matrix of Significant Biomarkers for All Participants

eTable 6. Sensitivity, Specificity, Positive Predictive Value (PPV), and Negative Predictive Value (NPV) for All Significant Biomarkers

eTable 7. Multivariable Logistic Regression Models for Predicting Reference Standard Autism Spectrum Disorder Diagnosis

eReferences

This supplemental material has been provided by the authors to give readers additional information about their work.

© 2024 Keehn B et al. *JAMA Network Open*.

eMethods. Description of Clinical Measures, Eye-Tracking Paradigms, and Eye-Tracking Data Processing

Participant Recruitment and Study Procedure

EAE Hubs receive referrals from regional PCPs for evaluation of children determined to be at increased likelihood for autism and then follow a standard evaluation protocol (i.e., DSM-focused developmental history and clinical interview, physical examination, and administration of an observational assessment of autism [Screening Tool for Autism in Toddlers; STAT[1]]).

Each EAE Hub referred a prospective, consecutive sample of children who had received an EAE Hub autism evaluation. This recruitment procedure allowed the study team to maintain diagnostic blindness and assess children with both autism and non-autism diagnoses (based on EAE Hub PCP diagnosis) without referral bias. During EAE Hub evaluations, PCPs (or a member of the EAE Hub clinical team; e.g., nurse or medical assistant) provided caregivers of children evaluated with a study brochure, brief verbal description of the study, and obtained signed consent to share contact information with the study team for recruitment and enrollment. Once enrolled, caregivers were sent an electronic survey to collect demographic information (i.e., child's race/ethnicity and caregiver/family income and education level) and a separate member of the study team (to maintain diagnostic blindness) collected the autism evaluation data (i.e., PCP autism/non-autism diagnosis and PCP diagnostic certainty). Participants were compensated with a gift card in the amount of $25 per hour of completed research evaluation (up to a maximum of $75).

Standardized Clinical Outcome Measures

A gold-standard research grade (reference standard) autism evaluation, including measures of developmental level, adaptive behavior, and autism symptom severity, was conducted by members of the study team, including a licensed clinical psychologist with expertise in autism diagnosis in toddlers and young children and clinical research technician (i.e., advanced graduate student or postdoctoral fellow).

Child Measures

Autism Diagnostic Observation Schedule, Second Edition (ADOS-2).[2] The ADOS-2 is a semi-structured standardized measure of social communication, play, and restricted and repetitive behaviors for individuals, ages 12 months and up, with varying developmental and language levels. Algorithm scores are compared with cutoff scores to yield a diagnostic classification (i.e., Autism, Autism Spectrum, or Non-spectrum; or Range of Concern for the Toddler Module). The Calibrated Severity Score (CSS) allows for examination of an individual's overall level of ASD-related symptoms in relation to those of other same-aged children diagnosed with ASD who have similar language skills. CSS scores were obtained following guidelines on the ADOS-2 protocol for Modules 1 and 2 and following recommendations in Esler et al.[3] for the Toddler Module. All ADOS-2 administrations were performed by an established ADOS-2 trainer or clinicians that had achieved research reliability with an established trainer.

Mullen Scales of Early Learning (MSEL).[4] The MSEL is a brief and reliable measure of cognitive ability in children from birth to 68 months of age, and includes five scales: Gross Motor, Visual Reception, Expressive Language, and Receptive Language. T-scores for each scale and an Early Learning Composite standard score are produced. To reduce floor effects, a developmental quotient (DQ) was calculated for verbal (Receptive and Expressive) scales and nonverbal (Visual Reception and Fine Motor) scales (i.e., DQ=age equivalent/chronological age x 100).

Caregiver Measures

Demographic and Background History Questionnaire. Brief caregiver-report questionnaire that collects information on child's race and ethnicity, family income, caregiver education level, and child medical history. In order to reduce the effects of missing race and ethnicity data, we combined these two variables into a race/ethnicity variable (see also Peltzman et al.[5] for discussion of the benefits of this approach).

Toddler ASD Interview for Caregivers. Standard caregiver interview questions assess for DSM-5[6] ASD criteria in toddlers. The interview was administered by the expert ASD specialist as part of the reference evaluation. Ratings are made based on caregiver report and observation of the child throughout the evaluation.

Vineland Adaptive Behavior Scales, Third Edition (VABS-3).[7] The VABS-3 is a parent/caregiver interview that assesses a child's adaptive skills across the domains of Communication, Daily Living Skills, Socialization, Motor Skills, and Maladaptive Behavior. Subdomain scaled scores, domain standard scores, and an Adaptive Behavior Composite standard score are derived from the measure.

EAE Hub PCP Measures

EAE Hub Diagnostic Data Form. EAE Hub PCP submits information on results of standard screening and observation tools administered at the EAE Hub evaluation, categorical autism diagnosis (autism; non-autism), non-autism diagnoses, and PCP diagnostic certainty (rated on a 5-point Likert scale; [1] Not certain at all – [5] completely certain).

Eye-tracking

Apparatus

The biomarker battery was presented using SR Research Experiment Builder 2.3.38 on a 17-inch LCD laptop computer (1920 x 1080). Participants were seated in a highchair or on caregivers lap approximately 80cm from the display. Eye movements and pupil diameter were recorded (500Hz; binocular) using an EyeLink Portable Duo remote eye-tracking system (SR Research, Ontario, Canada). Data collection occurred in seven EAE Hubs set within primary care practices (i.e., including six large health system group practices and one private practice).

Procedure

Participants first completed a five-point calibration and validation procedure while viewing an animated cartoon with sounds. Prior to the start of each block, a drift check and correction was completed. Data acquisition continued until the child became fussy or until sufficient data had been collected for each measure. Blocks were repeated as necessary to insure enough usable data were acquired.

Eye-Tracking Paradigms

Non-Social Preference. Adapted GeoPref task was modeled after Pierce et al.[8,9]; silent dynamic social and non-social videos (12° x 6.8°) were presented simultaneously on the left and right side of the screen (6.8° from center of screen) for 15 second blocks; four unique blocks were presented during the battery. Location of social and non-social videos was counterbalanced across blocks.

Resting Eye-Tracking. A dynamic central fixation that was presented on a black background for 30 second blocks. Four unique videos equated for luminance were used. Each video subtended approximately 4.2° x 4.2° degrees visual angle.

Gap-Overlap. Trials begin with a centrally presented dynamic fixation (3.5° x 3.5°). Once fixated (100ms; AOI: 7° x 7° square surrounding fixation), the central fixation remained onscreen for 900 to 1200ms. Next, a dynamic peripheral target (3.5° x 3.5°) identical to the central fixation was presented randomly either to the right or the left of the fixation stimulus at the eccentricity of 9.9°. The peripheral target remained onscreen until the target had been fixated or 3000ms had elapsed. In the gap condition, the central fixation disappeared 200ms prior to the

© 2024 Keehn B et al. *JAMA Network Open*.

onset of the peripheral target. In the overlap condition, the central fixation remained onscreen for the duration of the trial. Trials were presented in blocks of 8 trials.

Pupillary Light Reflex. Trials began with dynamic central fixation on a black background (3.9° x 3.9°) for a random duration between 1600 and 2400ms. Next, the display screen flashed white for 125ms, after which the screen returned to a black background with the same dynamic central fixation for 4000ms. Participants completed four trials in each block. Before each PLR trial, a 7000ms video clip of a rotating ball bouncing around the screen was displayed.

Visual Exploration. Between blocks measures described above children were shown 30 second videos of animated cartoons of nursey rhymes with sound (e.g., “row, row, row your boat”, “wheels on the bus”).

Preprocessing and Analysis

Non-Social Preference. Areas of interest (AOIs) were defined around left and right video locations (12.8° x 10°). Collapsed across all blocks, the total looking time within each AOI was determined and the percentage of looking within non-social AOI was calculated (non-social AOI duration / social + non-social AOI duration). To be included children needed to provide a total of 15 seconds of looking within AOIs.

Resting Eye-Tracking. A procedure similar to that described by Steiner and Barry[10] was used to calculate pupil diameter. Briefly, prior to data collection, an array of simulated pupils (2-10mm) was placed at multiple distances (550-800mm) from the eye tracker, using multiple pupil thresholds. These measurements were entered into a multiple linear regression and coefficients from this analysis were used to predict absolute pupil size for the current data set.

Thirty-second blocks were combined into a single time series. Next, for each eye, the following data were removed: 1) a 200ms buffer around missing data (e.g., blinks, period where participant rotates away from eye-tracking camera), outliers greater than 3 MAD, and time points where participant was not fixated within the central AOI (circle with 6.4° radius). Missing / excluded data were interpolated for gaps less than or equal to 1500ms, and segments of data less than three seconds were removed. Lastly, mean pupil size was calculated by averaging usable values for left and right eyes at each timepoint.

In addition, the distance between the eye tracker and the forehead of the participant was used to monitor participant movement. Specifically, the root mean square of the first temporal derivative of the distance measurement was used as a metric of overall head movement during resting pupil recording.

To be included children needed to provide a total of 15 seconds of usable pupil data while looking within the central AOI.

Pupillary Light Reflex. Pupil data were segmented 1500ms prior to and 4000ms after the flash. Processing pipeline was similar to Fish et al.[11]; this included a 100ms buffer around missing data (e.g., blinks) and removal of 1) outliers (3 SD) identified using moving window of 500ms, 2) large sample-to-sample pupil dilation changes (> 0.3mm), 3) extreme pupillary values less than 1mm or exceeding 10mm, and 4) pupil values for samples with gaze occurring outside the central fixation AOI (circle with 6.4° radius). Missing / excluded data were interpolated for gaps less than or equal to 500ms, and segments of data less than one second were removed. A 25-point moving average filter was then applied to the interpolated time series. First (velocity) and second (acceleration) order derivatives were then calculated. Latency of the PLR was defined by the acceleration minima between 110 - 570ms post-flash onset. Baseline pupil size (A_B) was determined for each trial by calculating the mean diameter for the 100ms interval prior to the latency measure, and the PLR amplitude was measured as the maximum constriction between 170 - 1450ms relative to PLR latency onset (A_M). Amplitude was calculated similar to Fan et al[12] ($[A_B^2 - A_M^2]/ A_B^2$).

Similar to Fish et al.[11], pupil time series were visually inspected to confirm accurate identification of both latency and amplitude measures. Furthermore, trials were excluded if latency, baseline, or amplitude window had more than 50% missing data. Participants were excluded if that had fewer than 3 usable trials after preprocessing. Median latency and amplitude were used.

© 2024 Keehn B et al. *JAMA Network Open*.

Gap-Overlap. Eye-movement data were analyzed from the onset of the peripheral target to the end of the trial. For each trial, the initial saccadic latency was include if: 1) fixation was nearest the central AOI, and thus the saccade was initiated from central fixation, 2) saccade was directed in the correct direction and saccade endpoint was nearest the peripheral AOI. No-shift trials were defined as trial without a saccade where participants were fixating the central stimulus at target onset and remained fixated for at least 1000ms. Trials were excluded if saccadic RT was less than 100ms. Participants were excluded if that had fewer than 3 usable trials for each condition after preprocessing. Saccadic reaction times (SRT) were determined by subtracting the time of onset of the saccade from the appearance of the target, and were used to calculate median SRT for gap and overlap conditions. No-shift percentage was calculated by dividing the number of no-shift trials by the number of usable trials for each condition. Although previous gap-overlap research has combined no-shift trials into SRT measures (by substituting maximum saccadic latency),[13,14] such approaches conflate latency and no-shift measures. Further, while no-shift percentage follows a similar pattern in autism (i.e., disproportionally longer RTs and higher percentages in overlap trials),[15] no-shift differences may provide unique information. Thus, gap-effect scores were created (overlap – gap) for both median SRT and no-shift percentages. To be include children had to have three usable trials for both gap and overlap conditions.

Oculomotor. For both the resting eye-tracking and visual exploration paradigms basic oculomotor metrics were extracted. Only fixations and saccades originating from paradigm-specific AOIs were included. Furthermore, fixations with durations less than 100ms were excluded and saccades with durations less than 10ms and longer than 1000ms or with peak velocities greater than 1500 °/s were excluded. For the Eyelink system, the onset/offset of blinks trigger a saccade parser, which classifies these events as blink saccades. Blinks were coded as blink saccades with a duration between 100 and 600ms. Oculomotor metrics examined include median fixation duration, rate of saccades, blink rate, saccade amplitude, duration, and peak velocity.

© 2024 Keehn B et al. *JAMA Network Open*.

eResults. Eye-Tracking Usable Data and Additional Regression Results

Usable Data

Acquisition of eye-tracking data was attempted on a total of 154 children; calibration / validation could not be completed with eight children (5.2%; ASD = 8; Non-ASD = 0), thus data from one or more paradigms were collected from 146 children. For the non-social preference test a total of 135 of 146 children (92.4%) provided usable data; two children fussed out before completing paradigm (ASD = 1; Non-ASD = 1) and 10 children did not provide enough usable data (ASD = 8, Non-ASD = 1).For the resting eye-tracking paradigm, 143 children received at least one block of resting eye-tracking and 113 provided usable data (79.0%). Of 30 children removed, 27 were excluded because they did not provide enough usable data (< 15s; ASD = 21; Non-ASD = 6) and 3 were excluded for non-standard sticker placement (ASD = 2, Non-ASD = 1). Of the 146 children, 103 were tested with the PLR paradigm. Of those tested, children completed an average of 8 trials (range: 4-18); fifty-seven children (55.3%) provided usable latency data and 51 (49.5%) provided usable amplitude data. For the gap-overlap paradigm, 136 of 146 children were tested. Of those children, 88 provided usable data for both gap and overlap conditions (3 usable trials per condition). For the visual exploration videos, 139 of 146 were presented with videos and 131 of 139 provided usable data. A total of eight participants (ASD = 7, Non-ASD = 1) provided less than 15s of looking time with the AOI.

For those that contributed at least one usable biomarker (n = 146), the number of missing biomarkers did not differ between autism and non-autism group, $\chi(5,146) = 5.18$, $p = 0.394$ (see eTable 1).

Biomarker Classification Accuracy

In addition to six significant biomarkers, six biomarkers did not significantly predict reference standard autism outcome (eTable 2). These include the reaction time measure of attentional disengagement, resting pupil size, and four oculomotor measure (saccade amplitude and duration) for both resting and exploration tasks.

© 2024 Keehn B et al. *JAMA Network Open*.

eFigure. Significant Eye-Tracking Biomarkers

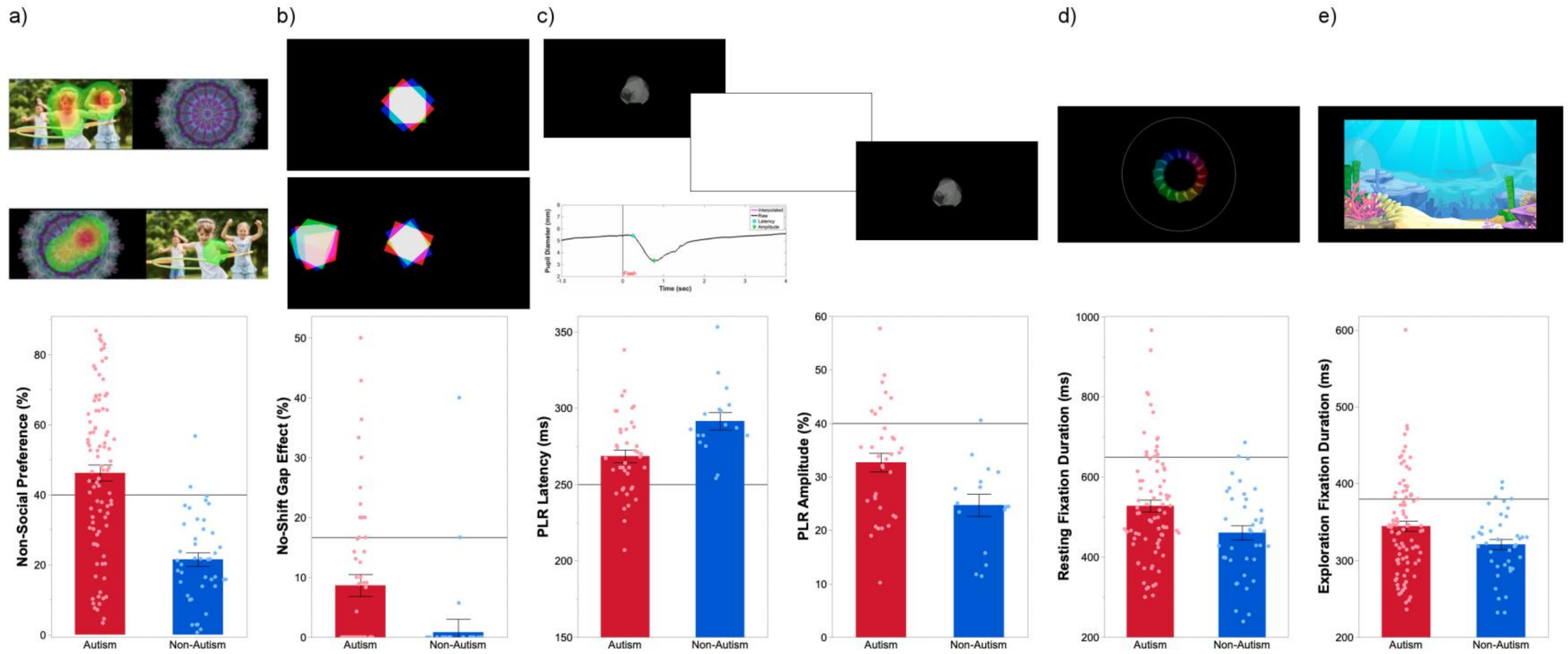

(a) Example of adapted GeoPref task with heat maps show social looking (top) and non-social looking (bottom) for a single block for two participants. (b) Example of gap-overlap paradigm with dynamic central fixation (top), which was displayed for 900-1200ms and overlap condition with dynamic central fixation and peripheral target both present (displayed until target fixated or 3000ms). (c) Stimuli for pupillary light reflex (PLR) with dynamic central fixation, brief flash, and central fixation. Example of PLR (bottom) with latency and amplitude. (d) Example of colorful dynamic central fixation used for resting eye-tracking task. Circular area of interest (AOI) used to include pupil and oculomotor measures. (e) Example of cartoon used to measure passive visual exploration. For bar

© 2024 Keehn B et al. *JAMA Network Open*.

graphs, error bars represent +/- 1 SEM, points reflect individual cases, and black horizontal line reflects 95% specificity cutoff used to create biomarker composite.

© 2024 Keehn B et al. *JAMA Network Open*.

eTable 1. Frequency and Percentage of Participants With Missing Biomarkers

Missing Biomarker(s)	Autism	Non-Autism
0	28 (27%)	12 (27%)
1	10 (10%)	5 (11%)
2	19 (19%)	6 (14%)
3	22 (22%)	16 (36%)
4	12 (12%)	3 (7%)
5	11 (11%)	2 (5%)

© 2024 Keehn B et al. *JAMA Network Open*.

eTable 2. Biomarkers Not Significantly Associated With Reference Standard Autism Spectrum Diagnosis

			Unadjusted Models						Adjusted Models				
**Predictors**	**Autism**	**Non-Autism**	***B***	**SE**	**OR (95% CI)**	**Wald χ^2**	**P-value**	**AUC**	***B***	**SE**	**OR (95% CI)**	**Wald χ^2**	**P-value**
**RT Gap-effect**	140.01 (54.20); 35.5 – 253.0	154.41 (58.60); 35 – 236.0	-0.005	0.74	1.00 (0.99 – 1.004)	1.09	0.297	0.58	-0.004	0.005	1.00 (0.99 – 1.005)	0.851	0.36
**Resting Pupil Diameter (mm)**	4.41 (0.65); 2.98 – 6.14	4.40 (0.48); 3.06 – 5.88	0.032	0.30	1.03 (0.57 – 1.87)	0.01	0.915	0.50	0.003	0.306	1.003 (0.55 – 1.82)	0.0001	0.99
**Resting Saccade Amplitude (°)**	1.52 (0.50); 0.88 – 3.54	1.64 (0.48); 0.78 – 3.45	-0.444	0.39	0.64 (0.30 – 2.36)	1.33	0.249	0.61	-0.432	0.393	0.65 (0.30 – 1.40)	1.208	0.27
**Resting Saccade Duration (ms)**	65.57 (23.82); 29.15 – 138.0	69.60 (28.30); 37.05 – 192.6	-0.006	0.008	0.99 (0.98 – 1.01)	0.67	0.414	0.53	-0.007	0.008	0.99 (0.98 – 1.01)	0.913	0.34
**Exploration Saccade Amplitude (°)**	3.28 (0.77); 2.03 – 5.38	3.16 (0.64); 1.66 – 4.74	0.240	0.269	1.27 (0.75 – 2.15)	0.79	0.372	0.52	0.301	0.277	1.35 (0.79 – 2.23)	1.180	0.28
**Exploration Saccade Duration (ms)**	59.16 (14.79); 36.48 – 121.34	62.26 (14.51); 44.02 – 95.41	-0.008	0.036	0.99 (0.92 – 1.06)	0.06	0.814	0.52	-0.003	0.037	1.00 (0.93 – 1.07)	0.009	0.92

Values in the Autism and Non-Autism columns are Mean (SD); range. B = unstandardized raw log odds coefficient; SE = estimated standard error of B; OR = odds ratio; CI = confidence interval; AUC = area under the curve. Adjusted models were adjusted for age and sex.

© 2024 Keehn B et al. *JAMA Network Open*.

eTable 3. Correlation Between Significant Eye-Tracking Measures and Autism Spectrum Disorder Severity, Developmental Levels, and Adaptive Skills

		All	Autism	Non-Autism
**Non-Social Preference**	**ADOS-2 CSS**	0.48* (134); 0.34 - 0.6	0.22 (92); 0.02 - 0.41	-0.1 (41); -0.39 - 0.21
	**MSEL ELCS**	-0.44* (134); -0.57 - -0.29	-0.26* (92); -0.44 - -0.06	-0.17 (41); -0.45 - 0.14
	**VABS-3 ABC**	-0.45* (134); -0.58 - -0.31	-0.31* (92); -0.48 - -0.11	-0.12 (41); -0.41 - 0.19
**NS% Gap-Effect**	**ADOS-2 CSS**	0.23 (76); 0 - 0.43	0.07 (53); -0.2 - 0.33	-0.13 (22); -0.51 - 0.3
	**MSEL ELCS**	-0.22 (76); -0.42 - 0.01	-0.16 (53); -0.41 - 0.11	0.1 (22); -0.32 - 0.49
	**VABS-3 ABC**	-0.27* (76); -0.47 - -0.05	-0.14 (53); -0.39 - 0.13	-0.19 (22); -0.56 - 0.24
**Resting Fixation Duration**	**ADOS-2 CSS**	0.28* (120); 0.11 - 0.44	0.19 (81); -0.03 - 0.39	0.08 (38); -0.24 - 0.39
	**MSEL ELCS**	-0.12 (120); -0.29 - 0.06	-0.04 (81); -0.25 - 0.18	0.12 (38); -0.2 - 0.42
	**VABS-3 ABC**	-0.1 (120); -0.28 - 0.08	-0.06 (81); -0.27 - 0.16	0.2 (38); -0.12 - 0.48
**PLR Latency**	**ADOS-2 CSS**	-0.3 (56); -0.52 - -0.05	0 (39); -0.31 - 0.31	0.06 (16); -0.44 - 0.52
	**MSEL ELCS**	0.1 (56); -0.17 - 0.35	-0.08 (39); -0.39 - 0.23	-0.49 (16); -0.79 - -0.02
	**VABS-3 ABC**	-0.07 (56); -0.33 - 0.19	-0.29 (39); -0.55 - 0.02	-0.49 (16); -0.78 - -0.01
**PLR Amplitude**	**ADOS-2 CSS**	0.38* (50); 0.11 - 0.59	0.06 (34); -0.28 - 0.38	0.36 (15); -0.16 - 0.73
	**MSEL ELCS**	-0.27 (50); -0.51 – 0	0.05 (34); -0.28 - 0.38	-0.18 (15); -0.62 - 0.35
	**VABS-3 ABC**	-0.14 (50); -0.4 - 0.14	0.05 (34); -0.28 - 0.38	0.15 (15); -0.37 - 0.6
**Fixation Duration**	**ADOS-2 CSS**	0.23* (130); 0.06 - 0.39	0.1 (90); -0.11 - 0.3	0.27 (39); -0.05 - 0.53
	**MSEL ELCS**	-0.07 (130); -0.24 - 0.1	0.05 (90); -0.16 - 0.25	0.02 (39); -0.29 - 0.33
	**VABS-3 ABC**	-0.1 (130); -0.27 - 0.07	-0.04 (90); -0.24 - 0.17	0.07 (39); -0.25 - 0.37

* $p < 0.0167$ (Bonferroni-adjusted threshold). Values are Pearson correlations (sample size); 95% confidence interval.

© 2024 Keehn B et al. *JAMA Network Open*.

eTable 4. Number of Participants Within Each Group Who Have 0 to 5 Positive Biomarkers

Number of Positive Biomarkers	Autism	Non-Autism
0	23 (23%)	34 (77%)
1	45 (44%)	10 (23%)
2	22 (22%)	0 (0%)
3	10 (10%)	0 (0%)
4	1 (1%)	0 (0%)
5	1 (1%)	0 (0%)

Number (percentage)

© 2024 Keehn B et al. *JAMA Network Open*.

eTable 5. Correlation Matrix of Significant Biomarkers for All Participants

	Non-Social %	No-Shift %	PLR Latency	PLR Amplitude	Resting Fixation Duration	Exploration Fixation Duration
**Non-Social %**	1					
**No-Shift %**	0.162 (77); -0.065 - 0.372	1				
**PLR Latency**	-0.040 (57); -0.298 - 0.223	0.124 (52); -0.155 - 0.384	1			
**PLR Amplitude**	0.053 (51); -0.226 -0.324	0.136 (46); -0.160 - 0.410	-0.490 (51); -0.6743 - 0.2475**	1		
**Resting Fixation Duration**	0.149 (119); -0.0323 - 0.320	0.182 (77); -0.044 - 0.390	-0.292 (57); -0.5138 - 0.0344*	-0.028 (51); -0.301 - 0.250	1	
**Exploration Fixation Duration**	0.053 (120); -0.127 - 0.230	0.036 (68); -0.204 - 0.272	-0.108 (49); -0.378 - 0.179	0.101 (44); -0.202 - 0.386	0.263 (110); 0.080 - 0.430**	1

**p* < 0.05, ** *p* < 0.001; *r* (n); 95% CI

© 2024 Keehn B et al. *JAMA Network Open*.

eTable 6. Sensitivity, Specificity, Positive Predictive Value (PPV), and Negative Predictive Value (NPV) for All Significant Biomarkers

Biomarker	Cutoff Value	Sensitivity	Specificity	PPV	NPV
**Non-Social %**	40%	0.613 (0.511 - 0.706)	0.952 (0.842 - 0.987)	0.966 (0.885 - 0.991)	0.526 (0.416 - 0.635)
**No-Shift %**	17%	0.241 (0.146 - 0.369)	0.913 (0.732 - 0.976)	0.867 (0.621 - 0.963)	0.339 (0.233 - 0.463)
**PLR Latency***	250ms	0.275 (0.161 - 0.428)	1 (0.816 - 1)	1 (0.741 - 1)	0.37 (0.245 - 0.514)
**PLR Amplitude**	40%	0.229 (0.121 - 0.39)	0.938 (0.717 - 0.989)	0.889 (0.565 - 0.98)	0.357 (0.23 - 0.508)
**Resting Fixation Duration**	650ms	0.183 (0.114 - 0.28)	0.949 (0.831 - 0.986)	0.882 (0.657 - 0.967)	0.356 (0.27 - 0.451)
**Exploration Fixation Duration**	380ms	0.264 (0.184 - 0.363)	0.925 (0.801 - 0.974)	0.889 (0.719 - 0.961)	0.356 (0.27 - 0.451)

*

© 2024 Keehn B et al. *JAMA Network Open*.

eTable 7. Multivariable Logistic Regression Models for Predicting Reference Standard Autism Spectrum Disorder Diagnosis

	Predictor	*B*	SE	OR (95% CI)	χ^2	P-value
**Non-Social Preference**	**Intercept**	-1.16	0.52	--	5.03	0.02
	**Hub Diagnosis**	0.68	0.27	3.93 (1.37 – 11.28)	6.46	0.01
	**Hub Certainty**	0.13	0.27	1.30 (0.44 – 3.83)	0.23	0.63
	**Hub Diagnosis * Certainty**	0.89	0.27	2.45 (1.44 – 4.16)	10.9	0.001
	**Biomarker**	0.06	0.02	1.06 (1.03 – 1.10)	14.23	< 0.001
**No-Shift % Gap-effect**	**Intercept**	0.5	0.34	--	2.26	0.13
	**Hub Diagnosis**	0.67	0.32	3.84 (1.08 – 13.61)	4.35	0.04
	**Hub Certainty**	0.29	0.33	1.77 (0.49 – 6.38)	0.78	0.38
	**Hub Diagnosis * Certainty**	1.02	0.32	2.78 (1.47 – 5.23)	10	0.002
	**Biomarker**	0.05	0.03	1.05 (1.001 – 1.11)	4.04	0.04
**Resting Fixation Duration**	**Intercept**	-2.04	1.12	--	3.36	0.07
	**Hub Diagnosis**	0.74	0.27	4.43 (1.53 – 12.86)	7.5	0.01
	**Hub Certainty**	0.5	0.27	2.72 (0.93 – 7.95)	3.32	0.07
	**Hub Diagnosis * Certainty**	1.11	0.28	3.03 (1.75 – 5.23)	15.74	< 0.001
	**Biomarker**	0.01	0	1.005 (1.001 – 1.01)	5.74	0.02
**PLR Latency**	**Intercept**	14.42	5.51	--	6.85	0.01
	**Hub Diagnosis**	0.83	0.44	5.30 (0.93 – 30.19)	3.52	0.06
	**Hub Certainty**	0.81	0.46	5.04 (0.84 – 30.17)	3.13	0.08
	**Hub Diagnosis * Certainty**	1.18	0.47	3.24 (1.30 – 8.08)	6.39	0.01
	**Biomarker**	-0.05	0.02	0.95 (0.92 – 0.99)	6.32	0.01
**PLR Amplitude**	**Intercept**	-3.55	1.73	--	4.23	0.04
	**Hub Diagnosis**	0.71	0.45	4.14 (0.70 – 24.41)	2.47	0.12
	**Hub Certainty**	0.29	0.46	1.79 (0.30 – 10.72)	0.41	0.52
	**Hub Diagnosis * Certainty**	1.55	0.53	4.73 (1.66 – 13.49)	8.45	0.004
	**Biomarker**	0.15	0.06	1.15 (1.03 – 1.30)	6.2	0.01
**Exploration Fixation Duration**	**Intercept**	-5.27	1.93	--	7.49	0.01
	**Hub Diagnosis**	1.07	0.28	8.52 (2.81 – 25.78)	14.36	< 0.001
	**Hub Certainty**	0.48	0.28	2.63 (0.89 – 7.80)	3.04	0.08
	**Hub Diagnosis * Certainty**	1.18	0.29	3.26 (1.85 – 5.75)	16.77	< 0.001
	**Biomarker**	0.02	0.01	1.02 (1.01 – 1.03)	9.45	0.002
**Composite Biomarker**	**Intercept**	0.87	0.29	--	8.96	< 0.001
	**Hub Diagnosis**	1.01	0.29	7.60 (2.42 – 23.94)	12.02	< 0.001
	**Hub Certainty**	0.10	0.29	1.23 (0.39 – 3.87)	0.13	0.72
	**Hub Diagnosis * Certainty**	1.05	0.29	2.89 (1.63 – 5.10)	13.22	< 0.001

© 2024 Keehn B et al. *JAMA Network Open*.

**Biomarker**	1.36	0.30	15.09 (4.68 – 48.65)	20.65	< 0.001

B = unstandardized raw log odds coefficient; SE = estimated standard error of B; OR = odds ratio; CI = confidence interval.

© 2024 Keehn B et al. *JAMA Network Open*.

eReferences

1. Stone WL, Coonrod EE, Turner LM, Pozdol SL. Psychometric properties of the STAT for early autism screening. *J Autism Dev Disord*. Dec 2004;34(6):691-701. doi:10.1007/s10803-004-5289-8

2. Lord C, Rutter M, DiLavore PC, Risi S, Gotham K, Bishop S. *Autism Diagnostic Observation Schedule, Second Edition (ADOS-2) Manual Part 1: (Modules 1-4)*. Western Psychological Services; 2012.

3. Esler AN, Bal VH, Guthrie W, Wetherby A, Weismer SE, Lord C. The autism diagnostic observation schedule, toddler module: standardized severity scores. *Journal of autism and developmental disorders*. 2015;45(9):2704-2720.

4. Mullen E. Mullen Scales of Early Learning. Circle Pines, MN: American Guidance Service; 1995.

5. Peltzman T, Rice K, Jones KT, Washington DL, Shiner B. Optimizing Data on Race and Ethnicity for Veterans Affairs Patients. *Military Medicine.* 2022;187(7-8):e955-e962. doi:10.1093/milmed/usac066

6. American Psychiatric Association, ed. *Diagnostic and Statistical Manual of Mental Disorders*. 5th ed. American Psychiatric Publishing; 2013.

7. Sparrow SS, Cicchetti DV, Saulnier CA. Vineland Adaptive Behavior Scales (3rd ed.). San Antonio, TX: Pearson; 2016.

8. Pierce K, Marinero S, Hazin R, McKenna B, Barnes CC, Malige A. Eye Tracking Reveals Abnormal Visual Preference for Geometric Images as an Early Biomarker of an Autism Spectrum Disorder Subtype Associated With Increased Symptom Severity. *Biol Psychiatry*. Apr 15 2016;79(8):657-66. doi:10.1016/j.biopsych.2015.03.032

9. Pierce K, Conant D, Hazin R, Stoner R, Desmond J. Preference for geometric patterns early in life as a risk factor for autism. *Arch Gen Psychiatry*. Jan 2011;68(1):101-9. doi:10.1001/archgenpsychiatry.2010.113

10. Steiner GZ, Barry RJ. Pupillary responses and event-related potentials as indices of the orienting reflex. *Psychophysiology*. Dec 2011;48(12):1648-55. doi:10.1111/j.1469-8986.2011.01271.x

11. Fish LA, Nystrom P, Gliga T, et al. Development of the pupillary light reflex from 9 to 24 months: association with common autism spectrum disorder (ASD) genetic liability and 3-year ASD diagnosis. *J Child Psychol Psychiatry*. Nov 2021;62(11):1308-1319. doi:10.1111/jcpp.13518

12. Fan X, Miles JH, Takahashi N, Yao G. Abnormal transient pupillary light reflex in individuals with autism spectrum disorders. *J Autism Dev Disord*. Nov 2009;39(11):1499-508. doi:10.1007/s10803-009-0767-7

13. McConnell BA, Bryson S. Visual attention and temperament: Developmental data from the first 6 months of life. *Infant Behavior & Development*. 2005;28:537-544.

14. Landry R, Bryson SE. Impaired disengagement of attention in young children with autism. *Journal of Child Psychology and Psychiatry*. Sep 2004;45(6):1115-22.

15. Keehn B, Kadlaskar G, Bergmann S, McNally Keehn R, Francis A. Attentional Disengagement and the Locus Coeruleus - Norepinephrine System in Children With Autism Spectrum Disorder. *Front Integr Neurosci*. 2021;15:716447. doi:10.3389/fnint.2021.716447

© 2024 Keehn B et al. *JAMA Network Open*.
