# Supplementary material for: Eye-Tracking Biomarkers and Autism Diagnosis in Primary Care
Source: JAMA Netw Open. 2024 May 14;7(5):e2411190. doi: 10.1001/jamanetworkopen.2024.11190 (PMC11094561; doi:10.1001/jamanetworkopen.2024.11190)
Supplement: Supplement 2. — Data Sharing Statement [file jamanetwopen-e2411190-s002.pdf]

Data Sharing Statement

Keehn. Eye-Tracking Biomarkers and Autism Diagnosis in Primary Care. *JAMA Netw Open*. Published May 14, 2024. doi:10.1001/jamanetworkopen.2024.11190

Data
Data available: Yes
Data types: Deidentified participant data
How to access data: Deidentified individual participant data has been shared as part of the National Institute of Mental Health Data Archive. https://nda.nih.gov/
When available: With publication

Supporting Documents
Document types: None

Additional Information
Who can access the data: Deidentified individual participant data has been shared as part of the National Institute of Mental Health Data Archive. https://nda.nih.gov/
Types of analyses: Data analysis purposes of shared data will also follow standard NIH NDA policies, as described at https://nda.nih.gov/faq.html#top
Mechanisms of data availability: NIH NDA data use requires a signed data use certification, described at https://nda.nih.gov/faq.html#dac.3
